# Estimation of the Biological Half-Life of Methylmercury Using a Population Toxicokinetic Model

**DOI:** 10.3390/ijerph120809054

**Published:** 2015-07-31

**Authors:** Seongil Jo, Hae Dong Woo, Ho-Jang Kwon, Se-Young Oh, Jung-Duck Park, Young-Seoub Hong, Heesoo Pyo, Kyung Su Park, Mina Ha, Ho Kim, Seok-Joon Sohn, Yu-Mi Kim, Ji-Ae Lim, Sang-Ah Lee, Sang-Yong Eom, Byoung-Gwon Kim, Kyoung-Mu Lee, Jong-Hyeon Lee, Myung Sil Hwang, Jeongseon Kim

**Affiliations:** 1 Molecular Epidemiology Branch, National Cancer Center, Goyang 410-769, Korea; E-Mails: joseongil@gmail.com (S.J); hdwoo@ncc.re.kr (H.D.W.); 2 Department of Preventive Medicine, College of Medicine, Dankook University, Cheonan 330-714, Korea; E-Mails: hojangkwon@gmail.com (H.J.K.); minaha@dku.edu (M.H.); limjiae@gmail.com (J.A.L.); 3 Department of Food and Nutrition, College of Human Ecology, Kyung Hee University, Seoul 130-701, Korea; E-Mail: seyoung@khu.ac.kr; 4 Department of Preventive Medicine, College of Medicine, Chung-Ang University, Seoul 156-756, Korea; E-Mail: jdpark@cau.ac.kr; 5 Department of Preventive Medicine and Heavy Metal Exposure Environmental Health Center, Dong-A University, Busan 602-714, Korea; E-Mails: yshong@dau.ac.kr (Y.S.H.); kimyumi@dau.ac.kr (Y.M.K.); medikim@dau.ac.kr (B.G.K.); 6 Molecular Recognition Research Center, Korea Institute of Science and Technology, Seoul 130-722, Korea; E-Mail: phs3692@kist.re.kr; 7 Advanced Analysis Center, Korea Institute of Science and Technology, Seoul 130-722, Korea; E-Mail: pks6475@kist.re.kr; 8 Department of Epidemiology and Biostatistics, School of Public health, Seoul National University, Seoul 151-742, Korea; E-Mail: hokim@snu.ac.kr; 9 Department of Preventive Medicine, College of Medicine, Chonnam National University, Gwangju 501-746, Korea; E-Mail: sohnsjx@chonnam.ac.kr; 10 Department of Preventive Medicine, School of Medicine, Kangwon National University, Chuncheon 200-701, Korea; E-Mail: sangahlee@kangwon.ac.kr; 11 Department of Preventive Medicine, College of Medicine, Chungbuk National University, Cheongju 362-763, Korea; E-Mail: esangy@cbu.ac.kr; 12 Department of Environmental Health, Korea National Open University, Seoul 110-791, Korea; E-Mail: kmlee92@knou.ac.kr; 13 Institute of Environmental Safety and Protection, NeoEnBiz Co., Bucheon 420-130, Korea; E-Mail: jhlee@neoenbiz.com; 14 Food Safety Risk Assessment Division, National Institute of Food and Drug Safety Evaluation, Cheongwon 363-700, Korea; E-Mail: hwang1963@korea.kr

**Keywords:** biological half-life, dietary exposure, methylmercury, one-compartment toxicokinetic model, population model

## Abstract

Methylmercury is well known for causing adverse health effects in the brain and nervous system. Estimating the elimination constant derived from the biological half-life of methylmercury in the blood or hair is an important part of calculating guidelines for methylmercury intake. Thus, this study was conducted to estimate the biological half-life of methylmercury in Korean adults. We used a one-compartment model with a direct relationship between methylmercury concentrations in the blood and daily dietary intake of methylmercury. We quantified the between-person variability of the methylmercury half-life in the population, and informative priors were used to estimate the parameters in the model. The population half-life of methylmercury was estimated to be 80.2 ± 8.6 days. The population mean of the methylmercury half-life was 81.6 ± 8.4 days for men and 78.9 ± 8.6 days for women. The standard deviation of the half-life was estimated at 25.0 ± 8.6 days. Using the direct relationship between methylmercury concentrations in blood and methylmercury intake, the biological half-life in this study was estimated to be longer than indicated by the earlier studies that have been used to set guideline values.

## 1. Introduction

Mercury is a widely distributed environmental toxicant and exists in several forms. Methylmercury (MeHg) is the most common form within living organisms, and it accumulates throughout the food chain [1]. Methylmercury is well-known for causing adverse health effects on the nervous system, liver, kidney, and reproductive organs of humans, and it primarily affects the central nervous system of the fetus, resulting in neurodevelopment toxicity [2,3,4,5]. The main source of methylmercury for humans is through the dietary intake of contaminated fish [6,7]. Thus, the intake of fish that is high in methylmercury is of great concern to childbearing women. However, fish is also a source of essential long-chain polyunsaturated fatty acids, which are important for brain development and functions [8,9]. Therefore, guidelines for a safe oral intake level of methylmercury should be established.

The U.S. Environmental Protection Agency (EPA) announced an oral reference dose (RfD) for methylmercury of 0.1 µg/kg bw/day [10]. The Joint Food and Agriculture Organization/World Health Organization Expert Committee on Food Additives (JECFA) established a provisional tolerable weekly intake (PTWI) value for methylmercury of 1.6 µg/kg bw/week, which is sufficient to protect the embryo and fetus from neurodevelopment toxicity [11]. The European Food Safety Authority (EFSA) reevaluated this reference value and set a tolerable weekly intake (TWI) of 1.3 µg/kg bw/week after considering the counteracting effects of omega-3 fatty acids in fish [12]. The point of departure values, such as a benchmark dose lower limit (BMDL) that was used to derive these guideline values, were generally calculated based on blood or hair mercury levels. Thus, it is necessary to convert blood or hair mercury levels to daily oral intake to derive the guideline value, and this conversion model has been used in a one-compartment model [13]. The elimination constant derived from the biological half-life of methylmercury in blood or hair is an essential part of the one-compartment model. The elimination constant used for the above three guideline values was 0.014 per day, which was calculated by the Study Report to Congress (MSRC) of the U.S. EPA [14] using the average values of four previous studies [15,16,17,18].

The estimation of the biological half-life has also been conducted in several previous studies [13,15,16,17,18,19,20,21]. However, the biological half-lives of methylmercury have been estimated differently, and uncertainty factors must be considered: the hair to blood ratios showed a wide variation [20]; only a small sample size of the relatively high levels of total mercury concentration was used; and total mercury levels were measured instead of methylmercury levels. Furthermore, a continuous exposure to a low dose could result in different values compared with a short-term exposure to a high dose.

In the present study, we used a population kinetic model with a large sample size representing a population of Korean adults to estimate the biological half-life of methylmercury. The direct relationship between the methylmercury concentration in blood and the daily methylmercury intake was used for these estimates.

## 2. Materials and Methods

### 2.1. Study Population

The study subjects were partly recruited from a cohort of the Korean Research Project on Integrated Exposure Assessment to Hazardous Materials for Food Safety (KRIEFS). The KRIEFS cohort consisted of 4867 subjects from various districts and age groups representing the Korean population. The age group was classified into 3 groups: adults, students, and babies with their parents. The subjects were divided into four groups according to the total mercury concentration: group I (p25–p50), group II (p50–p75), group III (p75–p95), and group IV (p95–p100). A hundred subjects were randomly selected from each group, and the majority of the selected populations were adults. Of the subjects in the adult group for our analysis (n = 305), with the exclusion of a subject with a missing value for dietary information (n = 1), a total of 304 adults (167 men and 137 women) aged 19 to 83 years old were finally selected. We collected demographic information including sex, age, and weight from all participants. All participants were provided a written informed consent form, and the study protocol was approved by the Institutional Review Board of the Dankook University Hospital (IRB protocol number DKUHIRB2010-04-0093). Written informed consent was obtained from all participants.

### 2.2. Methylmercury Assessment in Diet

A trained interviewer facilitated the 24-hour recall (24 h) interviews face-to-face, and another non-consecutive 24 h interview was conducted by telephone within one week to control for within-individual variation between June and August in both 2010 and 2011. After collecting the dietary data, we calculated the individual food intake using CAN-PRO 3.0 (Computer Aided Nutritional Analysis Program, The Korean Nutrition Society, Seoul, Korea). Among the 136 different food items reported, with 4410 food samples frequently and largely consumed in the Korean population, the 35 food items with 310 food samples showing a high mercury concentration were selected to assess the methylmercury concentration in food. A methylmercury concentration was detected in 18 food items, all of which were seafood. The remaining food items including 4 other seafood items were all below the limit of detection (LOD) (Supplemental Table S1). The methylmercury in the food was detected by gas chromatography with an electron capture detector, and the LOD of methylmercury was 6 μg/kg. The samples with no detected levels were assigned concentrations equal to half the LOD. The 2-day consumption data were linked to the 35 food items to calculate daily methylmercury consumption. The dietary exposure to methylmercury was estimated by multiplying the food consumption (g/day) by the methylmercury concentration (μg/g) for each day.

### 2.3. Methylmercury Assessment in Blood

Analysis of blood methylmercury concentration has been previously described [22]. Briefly, all of the blood was collected between 2010 and 2011 in Korea in anticoagulant-treated EDTA tubes and stored at −20 °C. The methylmercury concentrations in the blood of the study participants were analyzed with ethylate derivatization using headspace-gas chromatography-mass spectrometry, and the obtained LOD of methylmercury was 0.5 μg/L.

### 2.4. One-Compartment Toxicokinetic Model

In this paper, we used a one-compartment toxicokinetic (TK) model, as described by Swartout and Rice [23]. This model estimates the biological half-life of methylmercury by quantifying the relationship between the concentration of methylmercury in the blood and the daily intake of methylmercury. Suppose that the methylmercury concentration in the blood (MeHg_b_) is in a steady state, *i.e.*, the intake and excretion rates are in balance. The one-compartment model is given by the following equation:

$${\text{MeHg}}_{b}=\frac{d\times t_{1/2}\times \text{Abs}\times {\text{frac}}_{b}\times \text{w}}{\text{log}2\times {\text{V}}_{b}}$$

where *d* is the daily dietary intake (µg/kg bw/day), $t_{1/2}$ is the biological half-life of methylmercury (day), Abs is the gastrointestinal absorption fraction, ${\text{frac}}_{b}$ is the fraction of methylmercury in the blood, w is the body weight (kg) and ${\text{V}}_{\text{b}}$ is the blood volume of the body (L).

### 2.5. Statistical Model

We also used a Bayesian method for the population TK model of methylmercury. Let *y_i_* denote the methylmercury concentration in the blood and *d_i_* denote the daily dietary intake for the *i*th patient. The Bayesian population TK model was characterized as follows:

$$\log y_{i}=\log f\left(d_{i}\right)+\epsilon_{i},\epsilon_{i}\sim N\left(0,\delta^{2}\right)$$

$$\text{f}\left(d_{i}\right)=\frac{\left(d_{i}\times t_{\frac{1}{2},i}\times \text{Abs}\times {\text{frac}}_{\text{b}}\times {\text{w}}_{\text{i}}\right)}{\left(\text{log}2\times {\text{V}}_{\text{b}}\right)},$$

where $\epsilon_{i}$ is a normally distributed measurement error.

To estimate the parameters, we used the informative priors from the potential biological range given in Albert *et al.* [13], Stern [24] and the references therein as follows:

$$t_{1/2,i}\sim TN\left(\mu ,\tau^{2},0,\infty \right),$$

Abs ~ Unif (0.9,1),

$${\text{frac}}_{\text{b}}\sim \text{Unif }\left(0,0.1\right),$$

$${\text{V}}_{\text{b}}\sim \text{ Unif }\left(2.99,4.34\right)$$

where $\text{μ }\epsilon \mathbb{R},\tau^{2}>0,TN\left(\mu ,\;\tau^{2},a,b\right)$ denotes a truncated normal distribution with mean *µ* and variance *τ*^2^ on the interval (*a, b*) and Unif (*a, b*) represents a uniform distribution on (*a, b*). Additionally, we assigned priors for the hyper-parameters *µ* and *τ*^2^ as

$$\mu \sim TN\left(72,10.8^{2},0,\infty \right),$$

**Figure 1:**
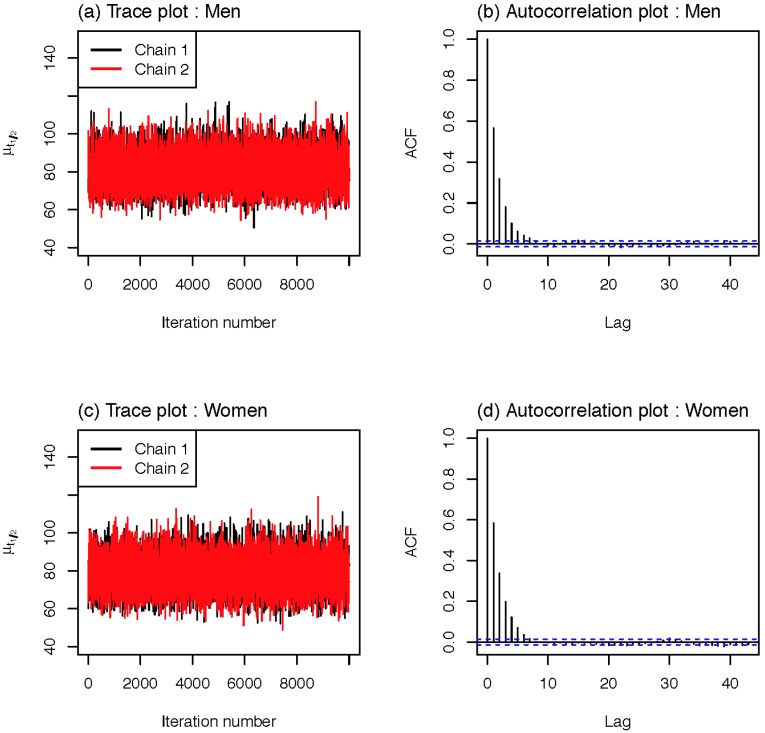
Diagnostic plots of MCMC outputs for population half-life; (**a**) trace plot and (**b**) autocorrelation plot for men, and (**c**) trace plot and (**d**) autocorrelation plot for women.

τ~Unif(10, 40)

and set a uniform prior between 0 and 100 for *σ* as a non-informative prior [25]. We used JAGS (http://mcmc-jags.sourceforge.net) version 3.3.0 for the Markov chain Monte Carlo (MCMC) simulation. In the MCMC simulation, we ran the Gibbs sampler for 60,000,000 iterations, with two chains at different initial values. For each chain, we saved every 5,000th iteration after a burn-in period of 10,000,000 samples was discarded, and we obtained 20,000 samples from the two chains. Based on an examination of trace plots and autocorrelation plots (Figure 1), there was efficient mixing. *i.e.*, trace plots do not represent irregularities and the autocorrelation lag is about 8 iterations.

## 3. Results

### 3.1. Characteristics of Study Population

Table 1 provides the descriptive statistics of the adult population included in this study. The total concentration of mercury and methylmercury in the blood was reported to be higher in the male population than the concentration in the female population, whereas the dietary intake per kilogram of body weight for men was comparable to that in the female population.

### 3.2. The Estimated Biological Half-Lives of Methylmercury

**Figure 2:**
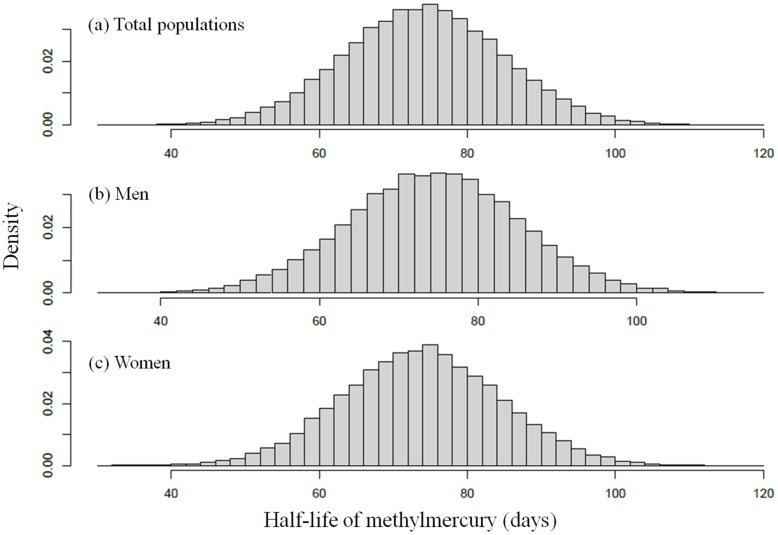
Population distribution for methylmercury half-life (years); posterior distribution for (**a**) total populations, (**b**) men, and (**c**) women.

We report posterior estimates calculated from the joint posterior distribution of model parameters for the one-compartment TK model in Table 2 and present the posterior distribution for the population mean (*μ*) of the methylmercury half-life in Figure 2. The population mean (*μ*) for the half-life of methylmercury was estimated at 80.2 days with a 95% credible interval of (64.0, 97.8). For males, this value was 81.6 days, with a 95% credible interval of (66.0, 98.8), and for females, the methylmercury population mean (*μ*) was estimated at 78.9 days, with a 95% credible interval ranging from 62.8 to 96.4 days, which was lower than that of the male population. The standard deviation (*τ*) of the methylmercury half-life *t*_1/2_ was estimated at 25.0 ± 8.6 days, indicating a large amount of between-person variability in *t*_1/2_.

### 3.3. The Reported Half-Life of Methylmercury

Table 3 presents the biological half-lives for methylmercury that were calculated in previous studies. The averages of the half-life values in blood ranged between 44.8 and 94 days, with minimum and maximum values of 35.1 and 155 days, respectively. The average half-life values in hair ranged between 65.4 and 102 days, with minimum and maximum values of 35 and 192 days, respectively.

## 4. Discussion

The estimation of methylmercury’s biological half-life from blood or hair is an essential part of the risk assessment of exposure to methylmercury. This paper used a one-compartment TK population model to estimate the half-life of methylmercury in blood. The estimated posterior mean of the population half-life was 80.2 days for all adults and 81.6 and 78.9 days for male and female adults, respectively. We also considered the inter-variability in the population, and the estimated standard deviation of the half-life was 25.0 days.

**Table 1:** Characteristics of the study population.

Variables	Total (n = 304)		Male (n = 167)		Female (n = 137)	
	Mean ± SD	Median	Mean ± SD	Median	Mean ± SD	Median
Age (years)	48 ± 14	50	47 ± 14	47	50 ± 14	51
Weight (kg)	66 ± 12	65	72 ± 11	72	60 ± 10	58
Total mercury in blood (µg/L)	8.69 ± 5.96	7.37	10.12 ± 6.9	8.33	6.94 ± 3.93	5.93
Methylmercury in blood (µg/L)	6.23 ± 4.31	4.98	6.97 ± 4.84	5.72	5.32 ± 3.36	4.17
Methylmercury intake from food (µg/kg bw/day)	0.05 ± 0.06	0.031	0.048 ± 0.056	0.031	0.053 ± 0.063	0.033

**Table 2:** Posterior estimates of model parameters.

Parameters	Total (n = 304)					Male (n = 167)					Female (n = 137)				
	Mean	SD	2.5%	50%	97.5%	Mean	SD	2.5%	50%	97.5%	Mean	SD	2.5%	50%	97.5%
*μ* (days)	80.2	8.6	64.0	80.0	97.8	81.6	8.4	66.0	81.4	98.8	78.9	8.6	62.8	78.6	96.4
*τ* (days)	25.0	8.6	10.8	25.0	39.1	25.0	8.6	10.8	25.0	39.2	25.0	8.6	10.8	25.0	39.2
Abs	0.955	0.028	0.904	0.956	0.998	0.955	0.028	0.904	0.958	0.998	0.954	0.028	0.904	0.956	0.998
V_b_	3.423	0.330	3.004	3.349	4.193	3.372	0.307	3.001	3.297	4.136	3.474	0.345	3.008	3.410	4.227
frac_b_	0.089	0.008	0.069	0.091	0.100	0.091	0.007	0.072	0.092	0.100	0.088	0.009	0.067	0.090	0.100
σ (µg/L)	1.388	0.108	1.191	1.388	1.601	1.459	0.081	1.310	1.454	1.631	1.318	0.082	1.170	1.314	1.490

*μ*: Population mean *t_1/2,_τ:* Population standard deviation for *t_1/2_*_._

**Table 3:** Biological half-life of methylmercury given in previous studies.

Reference	Sample Collection	Population/n	Exposure	Half-Life Measurement	Mean Intake	Concentration (Hair or Blood)	Half-Life/Mean (Days)
Miettinen *et al.* (1971) [16]	Hg/Blood	Finland, adults/6	Radiolabeled MeHg meal	No consideration of the baseline blood Hg concentration	0.3 (µg/kg/day)	−	49.8
Al-Shahristani and Shihab (1974) [18]	Hg/Hair at 8 to 12 mo.	Iraq (0.5–60 yr) /48	MeHg-contaminated grain	−	−	−	(Total) 72 (35–120), (90%) 65 (35–100)
Kershaw *et al.* (1980) [15]	Hg/Blood	Canada, male adults (19–44yr)/5	A single meal of fish (20 µg Hg/kg bw)	Consideration of the baseline blood Hg concentration	20.0 (18.1–21.8) µg/kg	−	52
Sherlock *et al.* (1984) [17]	Hg/Blood	UK, adults/20	High Hg concentrated fish, over 3 months	Consideration of the baseline blood Hg concentration	A 2.99, B 1.58, C 1.15, D 0.59, (µg/kg/day)	−	50
Smith *et al.* (1994) [19]	MeHg/Blood	U.S., male adults/7	Radiolabeled MeHg intravenously	MeHg remaining in the blood	−	−	44.8 (35.1–52.8)
Albert *et al.* (2010) [13]	Hg/ Hair at 12 wk/32 wk	French, pregnant women/125	FFQ conducted twice (seafood)	−	12 wk: 0.56, 32 wk: 0.67 (µg/kg/week)	Hair (µg/g): 12 wk: 0.82, 32 wk: 0.79	65.4
Yaginuma-Sakurai *et al.* (2012) [20]	Hg/ Blood and Hair	Japan, college students and graduates/27	Fish consumption (3.4 µg/kg/week)	With/without consideration of the baseline blood Hg concentration	3.4 (µg/kg/week)	Blood; 6.7 (3.2–19.8) ng/g, Hair; 2.3 (1.1–6.5) µg/g	Blood; 94 (58–155), Hair; 102 (60–192)

In previous studies, several methods have been used to calculate the biological half-life of methylmercury. A decline in the mercury concentration in blood or hair has been estimated after the administration of radiolabeled methylmercury [16,19] or the consumption of methylmercury contaminated foods such as fish and grain [15,17,18,20]. Another method used was the Bayesian inference method, which evaluated the hair mercury concentrations and methylmercury intakes using a food frequency questionnaire [13]. In early studies, the estimated biological half-life was relatively short [15,16,17], except for the estimations reported by Al-Shahristani & Shihab [18]. Miettinen *et al.* [16] estimated the half-life to be 49.8 days in a study of six Finnish adults who were fed a single meal of radiolabeled methylmercury. In another study, the half-life of methylmercury was estimated by observing the decline of mercury concentration in hair and was reported to be 72 days in an Iraqi population, but the distribution was bimodal [18]. The mean value from the main distribution containing 90% of the total subjects was 65 days and ranged between 35 and 100 days. Kershaw *et al.* [15] estimated a methylmercury half-life of 52 days in five Caucasian adults by observing the decline of the mercury concentration in blood following the consumption of a single meal of fish (approximately 20 µg Hg/kg bw); the prior blood mercury concentration was accounted for in this study. In the UK, the half-life of mercury was calculated using the relationship between methylmercury consumption from fish and the mercury concentration in blood [17]. Another study measured the methylmercury concentration in blood. Smith *et al.* [19] estimated the half-life to be 45.3 days based on the decline of methylmercury in blood among seven American male adults who were administered radiolabeled methylmercury intravenously. It was suggested that the methylmercury concentrations may have a shorter calculated half-life compared to the total mercury concentrations in blood due to the conversion of methylmercury to inorganic mercury [26]. Estimated methylmercury half-life was 50 days after considering the baseline blood mercury level.

The biological half-life for the calculation of an elimination constant to set guideline values was estimated at 50 days using the average values of the previous four studies [15,16,17,18], excluding that of Smith *et al.* [19]; this corresponds to an elimination constant of 0.014 per day [14]. The methods for measuring biological half-lives differed in the previous studies, including whether the mercury or methylmercury concentration was measured in the matrix. However, the estimated mean intakes of methylmercury in most of the above studies were considerably high. It has been reported that the intake dose and half-life of methylmercury are positively correlated [27]; however, recent studies with a relatively lower intake dose showed longer half-lives [13,20]. This could be the result of using a different specimen; for example, it appears that the half-life of methylmercury in hair is slightly longer than that in blood. Yaginuma-Sakurai *et al.* [20] estimated the half-life of methylmercury in both hair and blood specimens and reported that the half-life in hair was significantly longer than that in blood. Thus, it may not be appropriate to combine the results from different specimens. Another explanation for different half-lives is due to ethnic differences. The half-lives reported in the studies conducted in Asian countries were somewhat higher than those reported in other countries. The half-lives estimated in a population of long-term exposure to methylmercury may differ from those in a population of short-term, high concentration exposure, and the background level of methylmercury could affect the length of the half-life.

In the present study, the methylmercury half-life was estimated to be 80.2 days. The half-life in our study was higher than previous studies that used the mercury concentration in blood, but it was smaller than that of Yaginuma-Sakurai *et al.* [20], who reported relatively long half-lives in both hair and blood. This study suggested that subtraction of the baseline mercury level is not necessary; consequently, a higher half-life of methylmercury was derived. The advantage of our study is that we considered between-person variability using a large sample size. Albert *et al.* [13] also considered between-person variability, but their half-life standard deviation was large due to a small sample size. Furthermore, in contrast with the previous approaches that utilized the relationship between the mercury concentration in hair and dietary exposure, we estimated the biological half-life based on the association between the methylmercury concentrations in blood and the daily methylmercury intake. Therefore, our model did not contain a parameter reflecting the uncertainty of the hair to blood ratio, and, hence, our estimates of the population half-life may be more accurate than those in the previous studies. Studies have found that hair mercury concentration is not the best biological marker of methylmercury exposure because of external factors, such as dyeing, hair dressing, and hair permanents [28]. Although methylmercury generally accounts for a large amount of the total mercury concentrations [29], the ratio between methylmercury and total mercury might differ because of personal dietary patterns. Thus, our estimate of the biological half-life calculated based on the direct relationship between the methylmercury concentration in blood and methylmercury intake could be more accurate than those obtained in previous studies.

The present study has several limitations, including a one-compartment model. The limitations include that it did not allow realistic multi-compartment models, which are considered to fit several different sections of a body simultaneously, and it also did not use the quantities that represent individual characteristics such as sex, age, and body mass in the model. Therefore, the model can be extended to general models with parameters varying as functions of individual characteristics, which will offer more accurate estimates for the population half-life. The study population used in the present study was originally selected to investigate the correlation between methylmercury and total mercury among the entire cohort population. People with lower quartiles of total mercury concentration (below the 25th percentile) were excluded because very low levels of methylmercury might not be detected correctly. Thus, it could affect the results.

## 5. Conclusions

The population half-life of methylmercury was estimated to be 80.2 ± 8.6 days. The standard deviation of the half-life was estimated to be 25.0 ± 8.6 days. Our estimate of the biological half-life, based on the direct relationship between the methylmercury concentration in blood and methylmercury intake, was higher than those of the earlier studies that were used to set guideline values.

## Supplementary Files

Supplementary File 1
