# Supplementary material for: Estimation of the Biological Half-Life of Methylmercury Using a Population Toxicokinetic Model
Source: Int J Environ Res Public Health. 2015 Jul 31;12(8):9054–67. doi: 10.3390/ijerph120809054 (PMC4555264; doi:10.3390/ijerph120809054)
Supplement: Supplementary File 1 [file ijerph-12-09054-s001.pdf]

Estimation of the Biological Half-Life of Methylmercury Using a Population Toxicokinetic Model

Table S1. Methylmercury concentration in food (μg/kg).

Food	N *	Median	Min	Max
Shark	13	930.00	114.00	1679
Tuna	41	213.00	52.00	1059.59
Crab	10	166.95	46.00	187.00
Flatfish	29	83.74	<6.00	161.71
Hairtail	29	71.98	<6.00	128.05
Dried Squid	27	69.28	<6.00	120.72
Eel	15	60.14	7.00	82.88
Mackerel	20	51.38	<6.00	158.85
Loach	7	51.18	21.00	68.45
Whale	6	47.77	41.00	66.66
Dried anchovy	6	46.17	21.00	84.88
Pollack	6	43.80	<6.00	56.28
Canned Tuna	12	40.90	32.78	60.96
Yellow corvina	11	35.74	<6.00	70.34
Mackerel pike	36	35.24	<6.00	73.06
Squid	4	32.46	21.00	46.72
Fish cake	3	31.91	27.02	50.37
Mussel	5	<6.00	<6.00	46.00
Mandarin	4	<6.00	<6.00	<6.00
Small octopus	3	<6.00	<6.00	<6.00
Persimmon	2	<6.00	<6.00	<6.00
Pear	2	<6.00	<6.00	<6.00
Green pepper	2	<6.00	<6.00	<6.00
Oyster	1	<6.00	<6.00	<6.00
Dried laver	1	<6.00	<6.00	<6.00
Pork	1	<6.00	<6.00	<6.00
Soymilk	1	<6.00	<6.00	<6.00
Starch syrup	1	<6.00	<6.00	<6.00
Barley	1	<6.00	<6.00	<6.00
Powdered seasonings	1	<6.00	<6.00	<6.00
Spanish Mackerel	1	<6.00	<6.00	<6.00
Sugar	1	<6.00	<6.00	<6.00

Table S1. *Cont.*

**Food**	**N ***	**Median**	**Min**	**Max**
Salt	1	<6.00	<6.00	<6.00
Millet	1	<6.00	<6.00	<6.00
Sesame oil	1	<6.00	<6.00	<6.00

***** Analyzed sample numbers in each food items.

© 2015 by the authors; licensee MDPI, Basel, Switzerland. This article is an open access article distributed under the terms and conditions of the Creative Commons Attribution license (http://creativecommons.org/licenses/by/4.0/).
